# Supramolecular Adhesives Inspired by Nature: Concept and Applications

**DOI:** 10.3390/biomimetics10020087

**Published:** 2025-02-01

**Authors:** Abhishek Baral, Kingshuk Basu

**Affiliations:** 1 Department of Chemistry, Sister Nivedita University, DG Block, Action Area I, 1/2, Newtown, Kolkata 700156, West Bengal, India; 2 Department of Biological Chemistry, The Alexander Silberman Institute of Life Sciences, The Hebrew University of Jerusalem, Edmond J. Safra Campus, Jerusalem 9190401, Israel

**Keywords:** supramolecular adhesive, nature-inspired, self-assembly, non-covalent interaction, hydrogel, self-healing, wound dressing

## Abstract

Supramolecular chemistry, a relatively newly grown field, has emerged as a useful tool to fabricate novel smart materials with multiple uses. Adhesives find numerous uses, from heavy engineering to biomedical science. Adhesives are available in nature; inspired by them and their mechanism of adhesion, several supramolecular adhesives have been developed. In this review, supramolecular chemistry for the design and fabrication of novel adhesives is discussed. The discussion is divided into two segments. The first one deals with key supramolecular forces, and their implication is designing novel adhesives. In the second part, key applications of supramolecular adhesives have been discussed with suitable examples. This type of review casts light on the current advancements in the field along with the prospects of development.

## 1. Introduction

Non-covalent interactions play a key role in holding biological molecules. Proteins, nucleic acids, lipids, nutrients, and all other biologically relevant molecules recognize each other and interact using non-covalent forces. Non-covalent interaction is a collective term that includes several types of through-space molecular interactions, including hydrogen bonding (H-bonding), charge interaction, dipolar interaction, π-interaction, etc. The basic role of these interactions is to bind together structurally heterogeneous or homogeneous biomolecules to produce functional outcomes. Supramolecular chemistry relies exactly on these terms of non-covalent interactions, where these interactions are utilized in constructing three-dimensional superstructures. These “bottom-up” structures include fibers, vesicles, films, etc., with several functional properties, like self-healing, memory effects, adaptive structures, and mechanoresponsive properties.

Adhesion is a natural phenomenon that exists in every scale of life, from geckos’ feet to muscle adhesion on the marine bed. Bioadhesion is a special field of study that needs attention from several scientific disciplines. Intricate and extensive research has been conducted to identify the nature of bioadhesives and their gluing mechanism [1]. Understanding adhesion at the molecular level provides a strategic background for the fabrication of novel supramolecular adhesives. Wang and Stewart have discussed the role of dopamine moieties in the adhesion of *Phragmatopoma californica* (Fewkes) [2]. Moreover, dihydroxyphenylalanine (DOPA) is also relevant to muscle adhesion in the animal body [3], where the catechol group forms two hydrogen bonds using two *ortho*-hydroxyl groups. A lot of supramolecular adhesive materials have evolved based on catechol-containing motifs [4]. Catechol-conjugated polymers have proven to be effective in biomimetic adhesion [5] and medical adhesives [6]. To take this concept of bioadhesion, a new approach has been created to replace the hydrogen-bonding catechol group with host–guest complex-forming cucurbituril/ferrocene systems to fabricate underwater adhesives [7]. Another advantage of supramolecular adhesives is their reversibility in nature; they show nice stimuli responsiveness in the presence of certain physical or chemical influencers. Engineering multiple supramolecular interactions in a designed way has produced a number of stimuli-responsive adhesives [8]. Incorporating azobenzene, a known light-responsive moiety, has proven to be an effective way of fabricating stimuli-responsive dynamic interfacial adhesive [9]. Other non-covalent interactions such as π–π interaction, charged interaction, and hydrophobic effect have been utilized in fabricating novel supramolecular adhesives. These novel systems often hold much-advanced functionality, such as conductive healthcare hydrogels [10].

It is important to note that most of the non-covalent interactions that are utilized to design supramolecular adhesives are also responsible for producing hydrogels and organogels. In fact, in many cases, the hydrogels can be further fabricated to impart adhesion properties into the hydrogels [11,12]. The three-dimensional network of hydrogels can entrap water molecules up to a hundred to thousand times their weight, which is responsible for its biological soft tissue-like appearance. However, in many cases, this abundance of water gives rise to a boundary water layer that prevents direct interaction between the hydrogelator molecule and the substrate (like tissues) and negatively impacts the adhesion property of the hydrogel [13]. To overcome that, researchers have developed strategies to manipulate interactions within hydrogel cage-like structures to improve the adhesion nature of hydrogels [14]. One key property that a hydrogel/organogel should possess to become adhesive is the ability to continuously form newer bonds with different surfaces/biological tissues and also within its fragmented parts. Thus, adhesion is a step ahead of gelation as its constituent molecules not only bind themselves but are also capable of making bonds with other substances when kept in close contact. The strategies of forming adhesive gels include reversible catechol-quinone adhesion [15], hydrogen bonds [16], electrostatic [17], π–π interaction [18] and chemical linkages like amide bonds [19] or imine bonds [20]. Here, the current short review discusses some key examples of supramolecular adhesives along with a conceptual background of their molecular design and working principle. This kind of literature review may help pave the way for novel supramolecular adhesives with versatile utility.

## 2. Mechanistic Feature of Design

Supramolecular chemistry has helped scientists across several fields to produce smart materials with novel uses [21]. To construct a supramolecular material, one needs to be familiar with basic knowledge of non-covalent interactions. A detailed conceptual discussion of such intricate forces is out of the context of the current text. Therefore, we will discuss how these forces and interactions have helped to fabricate novel supramolecular adhesives.

### 2.1. H-Bonding

**Figure 1:**
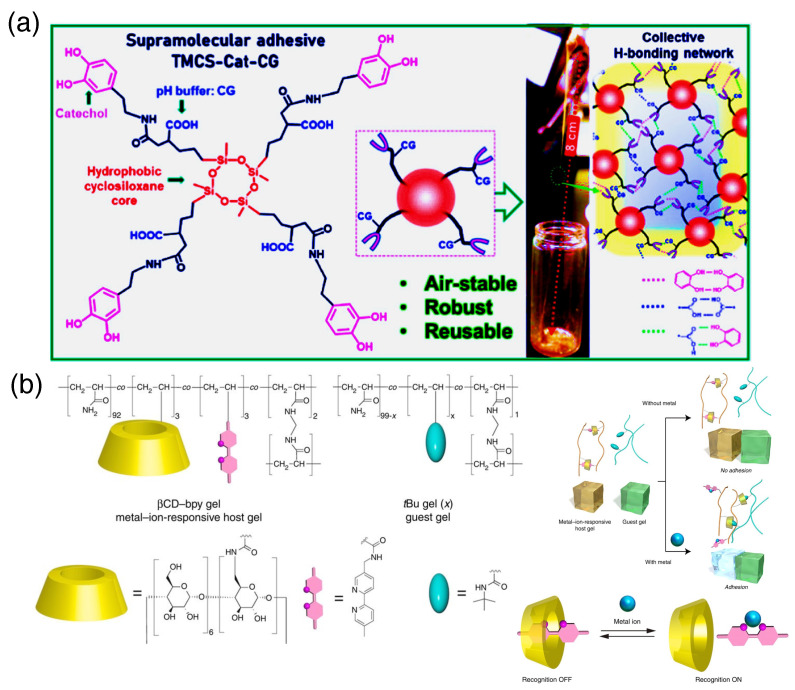
(**a**) Catechol-based molecular robust adhesives inspired by nature. Catechol forms a bidentate H-bonding network to form adhesion. H-bonding interaction and hydrophobicity make catechol groups protected from oxidation [28]. (Reproduced with permission from the American Chemical Society). (**b**) β-cyclodextrin (CD)- and 2,2’-bipyridyl (bpy)-based adhesive. The host–guest interaction provides stable adhesion, whereas the metal ion coordination site makes the adhesion dynamic. The dynamic nature also endows the gels with stimuli responsiveness (adapted from [29]).

H-bonding is the basic integrating force in the biological world. Supramolecular chemistry relies on this force to a great extent. The energy of most H-bonds lies in the borderline value where they can form and break fast enough to impose a dynamic nature in the biological world [22]. The most fascinating H-bond-based dynamic interaction is seen in DNA and RNA molecules. Based on this zipper-like interaction, several adhesive substances have been designed. Cheng et al. designed copolymers with adenine- (A) and thymine (T)-containing units to produce tunable adhesive and cohesive strength. At a 7 mol% concentration, adenine-containing polymers form distinct self-assembled structures, whereas thymine-containing polymers do not form any distinct morphology, even at higher concentrations. Interestingly, upon statistical admixture of both polymers, hydrogen bonds form between the donor and acceptor units, and thermodynamically stable, crosslinked structures are formed [23]. Later on, Gao and co-workers extended this idea but prepared adhesive hydrogels from the individual nucleobases adenine (A), guanine (G), cytosine (C), thymine (T) and uracil (U). Here, the authors introduced an acryloyl group with the purine or pyrimidine ‘N’ (which is attached to the sugar ring in DNA/RNA) and subjected it to polymerization with acrylamide in the presence of a radical initiator. This gave rise to polyacrylamide hydrogel being tackified by nucleobases that exhibit an adhesive property in plastics, glasses, rubber, stainless steel, and wood. This adhesive hydrogel powered by the strong H-bonding capability of the nucleobases holds promise for future biomedical applications as they can even be bound to physiological organs like the heart, liver, lung, and kidney, among others [24]. Urea-based moieties are also useful motifs due to the presence of a H-bond donor and acceptor group in the same –NH–CO–NH– backbone. Urea conjugated with morpholine and ureathane at its two ends was used as the starting material, and the ureathane units were copolymerized with PEG-like units to produce supramolecular polyurethane with mechanically recoverable properties at physiological temperature. These materials can fabricate in situ skin damage-repairing films when the experiment was carried out on pig skin [25]. Due to the excellent H-bonded ability of the urea moieties, they can also induce specific arrangements of the polymers for metal coordination to impart high stretchable potential. Zhang et al. designed urea-containing copolymers with tridendate 2,6-pyridinedicarboxamide that form rapid self-healing ultra-stretchable elastomers with high elongation (4000%) and good tear resistance extension (3500%) on conjugation with Fe(III) ions. Moreover, the addition of different concentrations of Fe(III) can regulate the viscoelastic properties of the supramolecular adhesive [26]. Natural sugars and acids are also good candidates for H-bond donors and acceptors and together can serve as stable adhesive hydrogels. Dong and co-workers reported mixing glucose, fructose, sucrose, or some other sugars with malic acid or citric acid at 2:1 or 1:1 ratios. The sugars and acids have multiple H-bonding sites that led to supramolecular polymerization and tough adhesion. Interestingly, the citric acid–sugar adhesive has more binding capacity towards the hydrophobic surface (like Teflon or polymethyl methacrylate) in comparison with malic acid adhesive. This probably comes from the higher number of –COOH groups in citric acid, which increases its H-bonding donation sites. The H-bonding network is so dense, that the adhesives exhibit high resistance towards organic solvents [27]. As described in the previous section, the catechol group is an excellent H-bond-forming group and serves as a supramolecular synthon for designing adhesive gels. Zhu et al. reported a hydrophobic tetramethylcyclotetrasiloxane-conjugated catechol-based oxidation-resistant adhesive material that can withstand robust conditions (Figure 1a) [28].

### 2.2. Host–Guest Interaction

Host–Guest interaction is a wide term where the supramolecular interactions can be varied in nature. In a host–guest complex H-bonding, π–π-stacking, electrostatic pairing, and hydrophobic interaction can occur in a concerted manner. A nice example of a host–guest interaction has been presented in Figure 1b [29] and will be discussed in the next section. Brush copolymers decorated with an azobenzene moiety form an excellent inclusion complex with β-cyclodextrin polymer to produce a glued polymer interface. This adhesion can hold more than 700 g cm^−1^ weight [30]. Hosts that can host more than one moiety can expand the scope of hydrogels. Recently, Scherman and co-workers developed a cucurbit[n]uril-threaded azobenzene (Az)-functionalized highly branched polymer hydrogel. This cucurbit[n]uril (CB) can further engulf another aromatic group, such as a phenyl ring (Ph) or another Az in its cavity. Therefore, when another polymer gel containing a second guest comes into contact with the previous one, a hetero-ternary complex is formed between CB-Az and Ph or Az. This adhesion has a very nice stress displacement pattern with strong adhesion energy values depending upon the composition of the gels. Interestingly, the stimuli responsiveness of the Az group makes the adhesion dynamic in nature and suitable for biomedical applications [9].

### 2.3. Metal Coordination

Metal coordination triggers supramolecular assembly when proper ligands are chosen. Recently, Stang and co-workers described a coordination polymer formed by crown ether compound, whose intricate balance between hydrophobicity and hydrophilicity is dictated by ligated metal ion [31]. In many cases, a preorganized assembly becomes stabilized and reinforced upon metal ion coordination [32]. Harada and co-workers exploited this particular concept to design a metal ion-responsive adhesive. They designed a polymer with both β-cyclodextrin (CD) and 2,2’-bipyridyl (bpy), which forms a stable hydrogel where bpy moieties become encapsulated by CD, resulting in no adhesion properties. Now, upon the addition of metal ions, the bpy becomes coordinated and expelled from the CD cavity, leaving free host CD segments. Therefore, this gel shows strong adhesion effects towards another foreign gel-containing potent host moiety: *^t^Bu* groups in the polymeric side chains (Figure 1b) [29]. Recently, Sun et al. demonstrated a metal ion-containing bio-based supramolecular adhesive with an adhesion strength of 14.6 MPa. They functionalized castor oil molecules with melevodopa to make a supramolecular coordination polymer with excellent record-high adhesion strength and cryogenic stability [33].

### 2.4. Electrostatic Interactions

**Figure 2:**
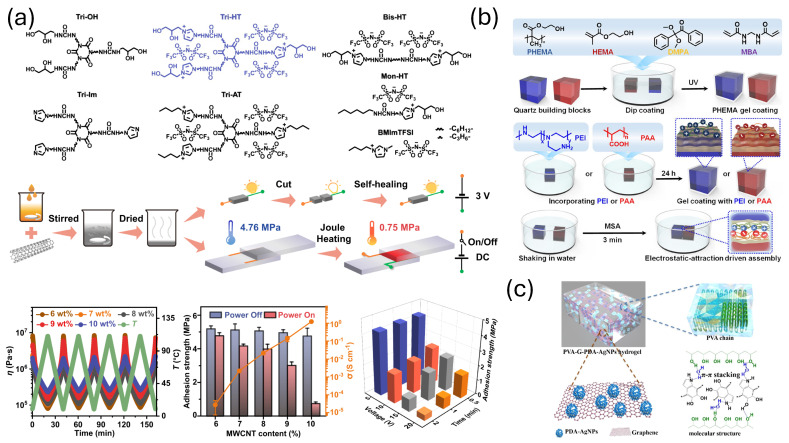
(**a**) Appending ionic liquid-like motifs can provide significant electrostatic interaction sites for an adhesive. PMBT, (poly(1–[2–methacryloylethyl]–3–methylimidazolium bis(trifluoromethane)-sulfonamide)), is a nice example of such a moiety where H-bond is hampered at the cost of electrostatic gain. The adhesion is stable at a higher temperature range (adapted from [35]). (**b**) Incorporating positive or negative charges into PHEMA-based adhesive on quartz (PEI to blue quartz; PAA to red quartz), and the molecular self-assembly produces a strong adhesion in the adhered solids [36]. (reproduced with permission from the American Chemical Society) (**c**) π–π stacking interaction between graphene and polydopamine provides adhesion in conductive composite hydrogels [37] (reproduced with permission from the American Chemical Society).

Electrostatic attraction and repulsion between opposite and same charges, respectively, dictates the stability of many supramolecular assemblies. Moreover, many times, the dielectric constant of the solvent involved in the assembly screens these attractive or repulsive Coulombic forces to produce optimum assembly conditions. In short, the gluing between molecules depends strongly on attractive or repulsive forces. An excellent example of this has been recently demonstrated by Zhu and co-workers. They designed a star-shaped random polymer PDAP (poly(diaminopyridine acrylamide)) and PThy (poly(thymine)), forming a heterocompatible strong electrostatic and H-bonding association. They involved a poly(ionic liquid)s segment, namely PMBT, (poly(1-[2-methacryloylethyl]-3-methylimidazolium bis(trifluoromethane)-sulfonamide)), within the structure to hamper H-bonding to some extent, which eventually promoted the adhesive properties of the gel on solid substrates [34]. Zhang and coworkers also developed a similar system with small molecular assembly. They meticulously hampered the H-bonded structure of a Tris-urea system by incorporating ionic liquid moiety, resulting in a strong adhesion character into the self-assembled material (Figure 2a) [35]. Complementary charges always attract each other; based on this, “flexible spacing coating” has been developed by Liu et al. with poly(2-hydroxyethyl methacrylate) (PHEMA) hydrogels. They created selective self-assembled surfaces with positive and negative charges to make naked-eye adhesion process as strong as $1018.1\pm 299.2\;{N/m}^{2}$ (Figure 2b) [36].

### 2.5. π–π Stacking

π-stacking is a kind of electron-dispersive interaction that binds two or more aromatic rings. This interaction holds important macromolecular structures such as nucleic acids and proteins in their native form. π–π stacking interaction between graphene and polydopamine has been exploited to produce an antibacterial self-adhesive gel. Interestingly, graphene is also a conducting material due to the long conjugated π surface, which introduces conducting properties in the gel. Moreover, the incorporation of Ag nanoparticles endowed the gel with antibacterial properties (Figure 2c) [37]. Many other examples of π-based small molecular self-assembled adhesives are available in the literature. Rowan and coworkers reported nice, healable supramolecular hydrogel based on a naphthalenediimide (NDI)–pyrene blend. NDI and pyrene are well-known π-accepting and π-donating groups and are thus often exploited to form stable donor–acceptor supramolecular complexes. This property was utilized in this work, where the authors synthesized one foldable polymer containing many NDI groups in the polymer chain and another polyurethane-based polymer having pyrene moieties as the end group. Then, they mixed the two polymers and the pyrene end group intercalates perfectly between the NDI groups of the foldable polymer to give rise to a regular structured tough supramolecular polymer blend [18]. Gu and coworkers have designed a polymer that contains dopamine-functionalized oxidized hyaluronic acid, adipic acid dihydrazide-modified hyaluronic acid, and aldehyde-terminated Pluronic F127 as polymer backbones. The presence of different type of functional ilities results in formation of a double network crosslinked gel having self-healing properties and shows improvement in skin regeneration by wound closure. The π–π interaction between the aromatic rings of the dopamine units plays a big role in the formation of the crosslinked hydrogel [38]. Nucleobase has wide π surfaces, and this property of the nucleobase in the form of methacryloylamidoadenine was exploited to prepare self-adhesive and antibacterial hydrogel. The polymer also contains chitosan in its backbone that gives mechanical stability and antibacterial character to the adhesive. The adenine derivative is responsible for the adhesion of the hydrogel with the tissues due to its ability to π stack and form H-bonds [39].

### 2.6. Hydrophobic Effects

**Figure 3:**
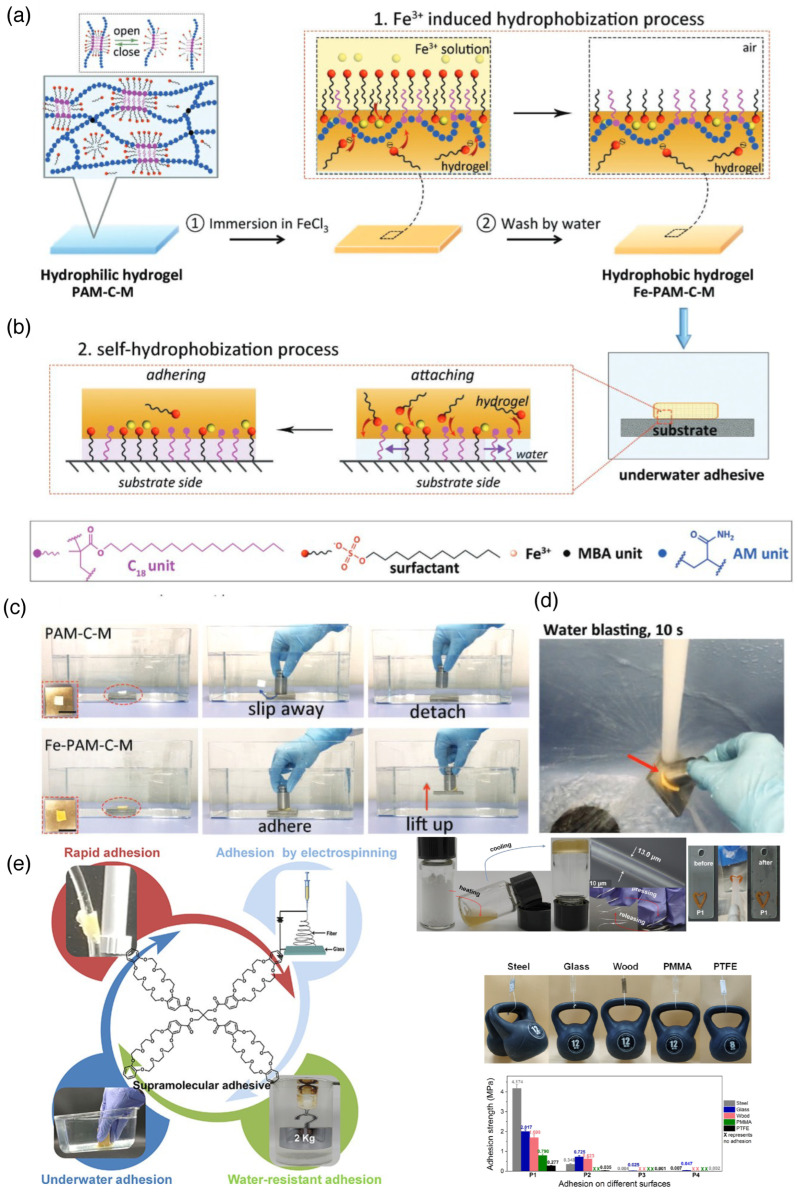
Underwater adhesive hydrogels. (**a**) Schematic illustration of the fabrication of the underwater adhesive hydrogels. The hydrogel (PAM-C-M) created from MBAA-crosslinked poly(acrylamideco-C18) was immersed in an aqueous Fe^3+^ solution followed by a water-washing process to obtain a hydrogel (Fe-PAM-C-M) with a hydrophobic surface. DI water was used, and MBAA is N,N′-methylenebisacrylamide. (**b**) Schematic illustration of the self-hydrophobization process for the formation of firm underwater adhesion between the hydrogel and substrate. When the hydrogel is compressed to achieve contact with the substrate underwater, the hydrophobic interactions form and grow at the interface and repel water away from the interface. (**c**) Demonstration of underwater adhesion. The as-prepared hydrophilic PAM-C-M hydrogel was nonadhesive and slipped away from the metal block surface underwater, while the hydrophobic Fe-PAM-C-M hydrogel firmly adhered to the metal block surface and was able to lift the block (200 g) up underwater. (**d**) Photograph showing that the adhesion between the hydrogel and substrate is strong enough to resist water blasting for 10 s (adapted from reference [40]). (**e**) Crown ether-appended hydrophobic moisture-proof adhesive, formation of glassy appearance upon heating and cooling with moldable shape formation properties (right side upper panel), and macroscopic adhesion with different substances with strong adhesion value (right lower panel) [41] (reproduced with permission from the American Chemical Society).

Hydrophobic interactions, where the non-polar parts of the molecules come close to each other to form aggregations, particularly in polar solvents like water, are less prominent in the context of supramolecular adhesives. This is because small molecules that behave as supramolecular adhesives have a lot of hydrophilic functional groups that participate in different non-covalent interactions, and relatively short hydrocarbon chains are not sufficient for generating significant hydrophobic impact. Hence, hydrophobic interaction is more common in the polymeric adhesives than their supramolecular counterparts. For example, Han et al. have used acrylamide and stearyl methacrylate (containing a hydrophobic long C18 chain) to induce free radical copolymerization in the presence of crosslinking initiator N,N′-methylenebis(acrylamide) and sodium dodecyl sulfate (SDS). This copolymer in the presence of Fe^3+^ ions creates a hydrophobic surface by generating water-resistant molecular bridges and enabling strong adhesion to various substrates. The hydrophobic C18 chain aggregates into ferric dodecyl sulfate and participate in hydrophobic associations that repel water molecules from the interface. They have also found that these hydrogels can also stick to the biological tissues in the presence of sweat, blood, and other body fluids [40]. Although hydrophobic effect is much weaker than others discussed earlier for supramolecular adhesives, sometimes this plays a big part in the stability of the adhesive material, particularly when the objective is to repel water. Thus, this effect is very crucial in designing underwater adhesive materials. Mitsuishi and coworkers have observed that the introduction of a tetramethylcyclotetrasiloxane (TMCS) hydrophobic core into a catechol-containing supramolecular adhesive reduces the propensity of oxidation of the ortho-OH groups of catechol, thus enhancing the adhesion efficiency and becoming an excellent candidate for marine adhesives (Figure 1a) [28]. Li et al. has covalently linked four dibenzo-24-crown-8 with four -OH groups of a pentaerythritol molecule to generate enough hydrophobicity (in spite of the presence of many ethereal oxygen in the crown-8 segments) to exhibit outstanding adhesion nature, even in high-moisture conditions. The successful adhesion properties of this molecule has been obtained in different surfaces like hydrophilic glass, iron polytetrafluoroethylene (PTFE), and poly(methyl methacrylate)(PMMA) (Figure 3) [41]. Long alkyl chains (C12 to C16) present in quaternary ammonium salts also participate in hydrophobic or solvophobic interaction with the H-bonded network formed by deep eutectic solvents (DESs) like choline chloride–urea. The interaction gives rise to supramolecular eutectogels that show adhesive nature at very low temperatures (like −196 °C of liquid N_2_) in addition to underwater adhesion properties [42].

## 3. Applications

### 3.1. Robust Supramolecular Adhesives

**Figure 4:**
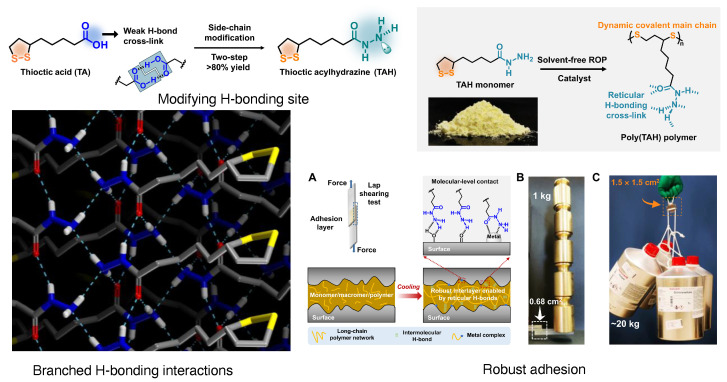
Modification of the carboxylic acid end with the acylhydrazine group increases the H-bonding interaction in thioctic acid, branched bonding interaction (lower left panel); (**A**–**C**) show the mechanism of robust adhesion (adapted from [43]).

Supramolecular adhesives are formed by weak non-covalent bonding interactions. These interactions are, in many cases, reversible and provide stimuli responsiveness in the resulting adhesions (as discussed in Section 2). To make supramolecular adhesion more robust, its dynamic properties needed to be screwed up. Crosslinking is the key process of holding together molecules in a supramolecular assembly; therefore, increasing and reinforcing the crosslinking is a key process in supramolecular adhesive design. Recently, Feringa and co-workers have solved this problem by a clever way by converting weak H-bonding carboxylic acid to strongly crosslinked H-bond-forming acylhydrazines. Moreover, it has a disulfide moiety, which provides a dynamic crosslinking network to the three-dimensional structure. They extended the scope of supramolecular robust adhesive to polymer materials by using this strategy (Figure 4) [43]. Along with strength, survival at robust conditions such as temperature, humidity, mechanical damage, etc., is also a prerequisite for adhesives. Molten adhesives, often used to bind two metal pieces, require high temperature resistance properties [44]. For typical supramolecular materials, high heat resistance is very difficult due to their weak and dynamic bonding properties. Increasing supramolecular crosslinking can solve this matter to a good extent. Poly-epoxy polymers are endowed with high supramolecular crosslinking; the incorporation of them with H-bonding 2-amino-4-hydroxy-6-methylpyrimidine by Sun et al. has produced remarkable outcomes. Moreover, flexible polymeric chains do not destroy the assembly in a molten state. The adhesive has been found to have good reusability with a high-temperature adhesion property of 10.2 MPa [45]. Damaging of the adhesion and the adhesive materials can lead to a severe impact on engineered structures. This problem has recently been circumvented by Liu et al. by appending a siloxane moiety with ureidopyrimidinone (Upy, a strong H-bonding motif, discussed in the next section). This siloxane also provides oil resistance properties to the adhesive coatings [46].

### 3.2. Stimuli-Responsive Adhesives

**Figure 5:**
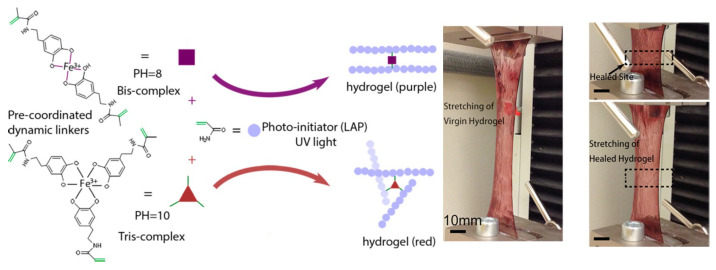
Photopolymerization of a Fe^3+^-coordinated catechol-based dynamic hydrogel. Healing of the stretched hydrogel holds potential promise for bioadhesion (right panel) [47] (reproduced with permission from the American Chemical Society).

The flexible nature of the non-covalent interactions often makes them sensitive to the surrounding environment. This imparts reversibility into the self-assembled system that can be exploited according to the requirement. Ma and coworkers have utilized mussel-inspired catechol–Fe^3+^ linkages as the key strategy in designing supramolecular hydrogels with self-healing properties. The catechol group of dopamine methacrylamide (DMA) form a complex with Fe^3+^. This preformed photo-polymerizable complex behaves as the monomer for UV light-induced polymerization in the presence of the radical initiator acrylamide. The coordination number of the ferric ion changes depending on the pH, which leads to different colored hydrogels at different pH. These pH and light-triggered hydrogels exhibit exceptional mechanical properties, stretching beyond 10 times their original length without rupture, and rapidly self-heal within 20 min after damage (Figure 5). This self-healing property can be exploited to obtain the adhesive nature. Moreover, the responsiveness of the material can be further seen as EDTA can induce the dissolution of the hydrogels by complexing out the Fe^3+^ ions [47]. The same Fe^3+^–catechol coordination is used to prepare hydrogel adhesive exhibiting NIR/pH responsiveness, which is utilized for wound dressing to cure skin infection arising from drug-resistant Staphylococcus aureus. Ureidopyrimidinone (UPy) has been used as a supramolecular self-assembling motif in this study, which provides the flexible backbone by forming a quadrupole hydrogen-bonding ability. This crosslinking is introduced by mixing poly(glycerol sebacate)-co-poly(ethylene glycol)-g-catechol prepolymer (PEGSD) and ferric ions to achieve the desired adhesive hydrogel. The dynamic nature of the Fe^3+^–catechol coordination together with the flexible H-bonding capacity of the UPy units resulted in the self-healing nature of the hydrogels. This self-healing can again be accelerated on NIR irradiation [48]. Zhao et al. has reported a conductive hydrogel suitable for wound dressing associated with diabetic foot ulcers. They also found an on-demand dissolution of the dressing in the presence of different type of stimuli like ultrasound and heating and also in the presence of chemicals like dopamine, vitamin B6, glucose, and fructose. The supramolecular hydrogel contains a H-bonded network provided by a mixture of polyvinyl alcohol, N-carboxyethyl chitosan, agarose, glycerin, and Ag nanowire together with sodium borate. Boron forms a strong bond with polyhydroxy compounds like glycerin and agarose to sustain the network structure together with the formation of H-bonds between the different components. The importance of this boron-oxygen structure is evident from the fact that all of the added chemicals that turn gel into sol contains multiple hydroxyl groups and thus disrupt the B-O bonds of the hydrogel [49]. As already mentioned earlier, the trans-cis photoisomerization nature of azobenzene is used by Scherman and coworkers to prepare adhesive materials based on the host–guest chemistry of cucurbit[8]uril. Irradiation of UV light over a ternary complex composed of CB[8], viologen, and azobenzene results in the expulsion of the azobenzene moiety from CB due to the cis geometry of azobenzene. In the process, the adhesive nature of the ternary complex has been disrupted. Thus, this study provides a stimuli-responsive (UV light) on-demand adhesion/de-adhesion material useful for designing tissue adhesives and materials for wound dressing [9]. Another stimuli-responsive group is anthracene, which undergoes dimerization on visible light (λ > 400 nm) and again comes back to a monomer on heating (70 °C). This property of a polyethylenimine-anthracene conjugate has been utilized to design reversible underwater glue, where adhesion strength can be tuned from about 51 kPa to 607 kPa [50].

### 3.3. Biologically Relevant Adhesives

**Figure 6:**
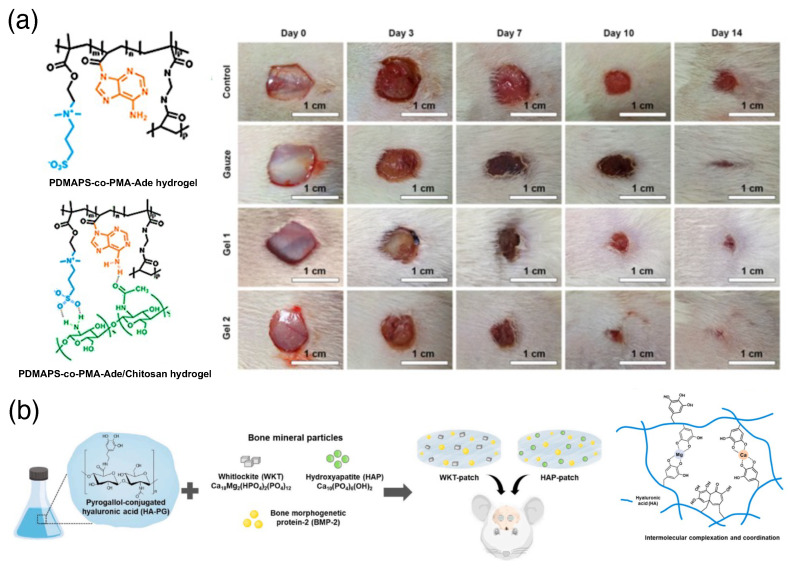
(**a**) PDMAPS-co-PMA-Ade/chitosan hydrogel as a wound dressing in a full-thickness skin defect. The left panel shows molecular structures and the right panel shows photographs of wounds treated by the control, gauze, PDMAPS-co-PMA-Ade (Gel 1), and PDMAPS-co-PMA-Ade/chitosan (Gel 2) hydrogel samples on days 0, 3, 7, 10, and 14 [39] (reproduced with permission from the American Chemical Society). (**b**) Illustration of preparing HA-PG hydrogel patches incorporated with inorganic particles (HAP, WKT) and BMP-2 and intermolecular complex formation through the coordination of oxidized PG moieties with ions released from HAP and WKT particles [55] (reproduced with permission from Elsevier).

In the biological and medical world, adhesives hold a prominent position in putting together morphologically and structurally heterogeneous organs [51]. Hagemann et al. used dopamine moiety as a bioinspired bioadhesive. They found better outcomes than commercially available bioadhesives with a significant nontoxic nature [52]. Crosslinked hydrogels with UPy synthone tethered with a poly(glycerol sebacate)-co-poly(ethylene glycol)-g-catechol prepolymer (PEGSD) chain with Fe^3+^ coordination act as a potential bioadhesive with in vivo blood clotting, skin incision/defect closure, and healing properties. Catechol–Fe^3+^ coordination was also exploited for producing stimuli-responsive properties in the hydrogel. They also studied the in vitro and in vivo efficacy of the gel to sterilize multi-drug resistant bacteria [48]. Bioadhesives with nucleobases/polymer/graphene oxide (GO) have been found to have human movement-sensing applications. The nucleobase hydrogen bonds produce good adhesion (on glass, metal, silicone rubber, and hogskin) and improve the mechanical performance of the gel phase upon muscle movement. The incorporation of nucleobase has been found to enhance the adhesion force on hogskin 2.5 times more than the native polymer gel [53]. One of the most important features of these supramolecular adhesives is their role in wound dressing/healing, and some of them are discussed in earlier sections [38,48,49]. The wounds can be due to normal skin tear (Figure 6a) [39], multidrug-resistant bacterial infection [48], or diabetic foot ulcers [49]. Skeletal repair is another key problem encountered by medical professionals. Lei and coworkers have reported a muscle-adhesive injectable hydrogel consisting of polypyrrole/polydopamine, poly(citrate glycol) polyethylenimine, and Pluronic F-127 terminated with dibenzaldehyde and carboxymethyl chitosan. The crosslinked hydrogel promotes the regeneration of skeletal muscles and improves myogenic differentiation [54]. Bone tissue regeneration with adhesive supramolecular gels has been carried out by using a pyrogallol-conjugated hyaluronic acid hydrogel. Inorganic materials like hydroxyapatite or whitlockite were incorporated into the hydrogel to form a hybrid nanocomposite. This nanocomposite was then mixed with bone morphogenetic protein-2 and applied on mouse model as a hydrogel patch that leads to bone regeneration at the defected site. The electrostatic interactions between inorganic particles from hydroxyapatite or whitlockite and bone morphogenetic protein-2 ensure the slow and sustained release of the protein from the hydrogel matrix. The ortho-hydroxy groups of pyrogallol bestow the hydrogel an adhesive nature like DOPA so it can stick to the bones without any glue (Figure 6b) [55]. Adhesive hydrogel has been also applied to cure neural tissue arising from spinal cord injury. A Schiff base reaction between the aldehyde-modified hyaluronic acid and adipodihydrazide-modified hyaluronic acid led to the formation of a hydrogel and adhesive nature that was imparted by modifying the hydrogel with an adhesive peptide PPFLMLLKGSTR. Then, human mesenchymal stem cell (HMSC)-derived exosomes were immobilized on the adhesive hydrogel to achieve 3D adhesion over the injury site. This ensures the sustained release of the exosomes, leading to neural tissue regeneration [56].

## 4. Advantages, Current Challenges, and Prospects

So far, we have discussed the key features of supramolecular adhesives, and a common advantage of supramolecular adhesives that has emerged is their stimuli responsiveness. Most of the supramolecular adhesives are dynamic and have proven effective in producing bioadhesives with multi-stimuli responses [57]. Moreover, including several additive properties like electronic conductivity in supramolecular adhesives is relatively easy due to their wide scope of functionalization [58]. Gao et al. have designed and fabricated an anion-coordinating molecular moiety that can form a stable adhesive gel in the presence of a phosphate anion. Moreover, due to the presence of extended conjugation and the ion-based helical bonding chain, the adhesive shows excellent electrical conductivity [59]. Recently, Liu et al. demonstrated a tricky way to design underwater adhesives. They designed a nucleobase-functionalized polymer that can form organogel in DMSO. Upon immersing this gel-based adhesive underwater, water–DMSO exchange leads to the formation of a robust adhesion with manifold enhancement of adhesion properties compared with the organogel [60]. It is worth mentioning here that this type of dynamic adhesion property is very hard to achieve using conventional adhesive materials.

Another aspect of supramolecular systems is their bio-adaptability. Supramolecular adhesives, formed by small molecules, are often degraded in biodegradation conditions. This degradability makes them advantageous over conventional adhesives. Surgical adhesives are nice examples where the biodegradation of the adhesives is very important. Recently, Guo et al. demonstrated a bionic strategy to prepare a biodegradable surgical adhesive using ε-polylysine decorated with gallic acid and polyethylene glycol diacrylate [61]. Other than this, biodegradable structural adhesives are more environmentally sustainable [62] than their conventional counterparts [63], which also make them advantageous for sustainable use.

Despite several advantages, there is a long way to go to reach the optimum outcome for supramolecular adhesions. The robustness, as discussed in the earlier section, has only reached up to a few M Pa in value, whereas conventional adhesives have reached 70 M Pa adhesion strength in ambient temperature [64]. Researchers need to put more emphasis on strength. The structure–property relationship of supramolecular adhesives needs to be investigated more to produce strong adhesion with a nice dynamic nature. There is definitely more room here available for investigation. Cost effectiveness is another key factor for the large-scale production of adhesives. Most of the supramolecular moieties need rigorous synthetic strategies and, therefore, are not very viable from the industrial-scale perspective. More effort is needed for cost cutting, such as by recycling the adhesive materials. Recently, Mulcahy et al. have discussed the scope of the recyclability of supramolecular adhesives in their review [65].

## 5. Conclusions

Understanding the basis of natural adhesion and applying it using supramolecular chemistry has produced significant outcomes in novel adhesive production. Scientists from different fields have investigated and created several state-of-the-art adhesives utilizing the basis of natural adhesion. Understanding supramolecular forces, including H-bonding, electrostatic interaction, π–π stacking, etc., and utilizing them to design novel adhesives has proven effective in producing both robust and dynamic adhesives. Dynamic adhesion is useful for stimuli-responsive adhesion and reversible adhesion, whereas robust adhesion needs more and more crosslinked systems with stronger adhesion. An amalgamation of both of these concepts has produced a lot of adhesives useful for biology and engineering science.

## Abbreviations

The following abbreviations are used in this manuscript:

Az	Azobenzene
bpy	2,2’-bipyridyl
CB	Cucurbit[n]uril
CD	Cyclodextrin
DES	Deep eutectic solvents
DMA	Dopamine methacrylamide
DOPA	Dihydroxyphenylalanine
GO	Graphene oxide
PAA	Polyacrylic acid
PDAP	Poly(diaminopyridine acrylamide
PEGSD	Poly(glycerolsebacate)-co-poly(ethylene glycol)-g-catechol
PEI	Polyethyleneimine
PHEMA	Poly(2-hydroxyethyl methacrylate)
PMBT	Poly(1-[2-methacryloylethyl]-3-methylimidazolium bis(trifluoromethane)-sulfonamide)
PMMA	Poly(methyl methacrylate)
PTFE	Polytetrafluoroethylene
pThy	Poly(thymine)
SDS	Sodium dodecyl sulfate
tBu	tert-butyl
TMCS	Tetramethylcyclotetrasiloxane
UPy	Ureidopyrimidinone
